# NSFC Health Research Funding and Burden of Disease in China

**DOI:** 10.1371/journal.pone.0111458

**Published:** 2014-11-04

**Authors:** Gelin Xu, Zhizhong Zhang, Qiushi Lv, Yun Li, Ruidong Ye, Yunyun Xiong, Yongjun Jiang, Xinfeng Liu

**Affiliations:** Department of Neurology, Jinling Hospital, Nanjing University School of Medicine, Nanjing, Jiangsu Province, China; Huazhong University of Science and Technology, China

## Abstract

**Background:**

Allocation of health research funds among diseases has never been evaluated in China. This study aimed to examine the relationship between disease-specific funding levels of National Nature Science Foundation of China (NSFC), the main governmental resource for health research in China, and burden of disease.

**Methods:**

Funding magnitudes for 53 diseases or conditions were obtained from the website of NSFC. Measures of disease burden, mortality, years of life lost (YLLs) and disability-adjusted life years (DALYs), were derived from the Global Burden of Disease Study 2010. The relationship between NSFC funding and disease burden was analyzed with univariate linear regression. For each measure associated with funding, regression-derived estimates were used to calculate the expected funds for each disease. The actual and expected funds were then compared. We also evaluated the impacts of changes of disease burden metrics since 1990, and differences from the world averages on NSFC funding.

**Results:**

NSFC health research funding was associated with disease burden measured in mortality (R = 0.33, P = 0.02), YLLs (R = 0.39, P = 0.004), and DALYs (R = 0.40, P = 0.003). But none of the changes of mortality (R = 0.22, P = 0.12), YLLs (R = −0.04, P = 0.79) and DALYs (R = −0.003, P = 0.98) since 1990 was associated with the funding magnitudes. None of the differences of mortality (R = −0.11, P = 0.45), YLLs (R = −0.11, P = 0.43) and DALYs (R = −0.12, P = 0.38) from that of the concurrent world averages were associated with the funding magnitudes. Measured by DALY, stroke and COPD received the least funding compared to expected; while leukemia and diabetes received the most funding compared to expected.

**Conclusion:**

Although NSFC funding were roughly associated with disease burden as measured in mortality, YLLs and DALYs. Some major diseases such as stroke were underfunded; while others such as leukaemia were overfunded. Change of disease burden during the last 20 years and country-specialized disease burden were not reflected in current allocation of NSFC funds.

## Introduction

In recent years, China is pouring cash into science and technology faster than its economy has expanded [1]. The total research and development (R&D) expenditure in China has increased by 23% annually on average over the past decade [2]. The central government's budget on science and technology reached 267.4 billion yuan renminbi (RMB), or US $43.6 billion, in 2014 [2]. Consistent with this trend, the governmental funding for health research is also increasing. Although still dwarfed by that of US (US $ 3.1 billion for National Natural Science Foundation of China (NSFC) against US $ 30 billion for US National Institute of Health (NIH) in 2014), China's governmental funds, principally NSFC, are becoming an important supporter for health research in the world [3].

However, the allocation of research funds among diseases, and its rationality has never been evaluated in China. The disbursement of grant money has been criticized for lack of transparency, and funding tends to go to eminent scientists and safe projects [4], [5]. There are short-term (annual) and long-term (5 years) national priorities for health researches, but they are merely results of government decisions based on politic will, public opinions, extemporaneous expert comments and immediate experience from western countries, rather than on systematic analysis of China's own disease burden. Considering the limited resources, it is important for China to optimize the process for health fund allocation with disease burden as a reference, as did in USA and other countries [6]–[8].

NSFC is a centre goverment agency specialized in administrating basic research funds, and it is the main source for the health research funds. Utilizing the design and methods in previous studies for evaluating association of NIH funding level and disease burden in USA [6], [7], this study evaluated the relationship between NSFC funding levels and measures of disease burden. We also discussed the differences of health research system in China and USA and the possible factors influencing the health fund allocation among diseases.

## Methods

The budgets for NSFC are determined at least one year before each fiscal year, and some major research plans are made even five years ago. To reflect this time lag, data of 2010 disease burden and 2012 NSFC funding were chose to evaluate the relationship between disease burden and magnitude of research funding. Diseases were included in this study if 1) they were defined in *International Classification of Diseases, 9^th^ revision, Clinical Modification (ICD-9-CM);* 2) their funding levels of NSFC were available; and 3) their measures of the burden of disease were available in Global Burden of Disease (GBD) Study 2010 [9].

### Data Sources

NSFC has eight departments responsible for funding researches in different scientific subjects: mathematics, chemistry, life science, geoscience, engineering and material science, information science, management science and health. Before 2010, health research was included in the life science department, which also involved specialties of biology, botany, ecology, zoology, forestry, microbiology, immunology, psychology, agronomy and veterinary medicine. In 2010, health research was separated from life science department as a separate department.

The health department of NSFC has a similar framework to NIH, but with fewer “institutes” (currently eight compared with 27 institutes and centers in NIH), and endued with less functions. Applicants submit their protocols to different institutes according to target disease. For example, No.1 institute is responsible for diseases in circulatory, respiratory, blood and digestive systems. No.2 institute is responsible for disease in urinary, genital and endocrine systems, and diseases of ophthalmology, otolaryngology and dentistry. No. 3 institute is responsible for diseases of neurology and psychology. No.4 institute is responsible for diseases of skin, locomotor system and injury. No. 5 institute is responsible for cancer. No. 6 institute is responsible for communicable diseases and occupational diseases. No. 7 institute is responsible for pharmacology and pharmaceutics. No. 8 institute is responsible for Traditional Chinese Medicine.

Data concerning the funding levels for specific diseases or conditions in 2012 fiscal year were obtained from the NSFC website (http://isisn.nsfc.gov.cn/egrantweb/). The funding data were updated annually by NSFC. Data concerning disease burden were derived from GBD Study 2010 [9].

Three measures were chose to reflect disease burden from the GBD Study 2010: mortality, years of life lost (YLLs), and disability-adjusted life years (DALYs). In GBD Study, YLLs are calculated by multiplying the number of deaths in each age group by a reference life expectancy at that age. YLDs are calculated from the prevalence of a sequela multiplied by the disability weight for that sequela. Disability weights are based on surveys of general population. DALYs are the sum of YLLs and YLDs [10].

Due to the rapid population ageing and widespread westernized lifestyle changing, there is a remarkable change of disease spectrum in China in recent 20 years. These trends may have influenced the allocation of research resources, because some disease may become more, while others less prevalent. We, therefore, evaluated the impacts of changes of mortality, YLLs, DALYs between 1990 and 2010 on disease funding. Data of disease burden, mortality, YLLs and DALYs for China in 1990, were also retrieved from GBD Study [9].

Some diseases are more prevalent, and pose an extra burden in China compared to the world average level. These diseases deserve extra research resource to accelerate improvements in prevention and management. Therefore, we evaluated the differences of mortality, YLLs and DALYs of each disease between China and the world average level. Data of mortality, YLLs and DALYs for the world average level in 2010 were retrieved from GBD Study [11]–[13].

### Statistic Analysis

Univariate linear regression was performed to assess the relationship between funding levels and disease burden metrics. For each measure that had a significant association with funding, regression-derived estimates were used to calculate the expected NSFC funding for each disease. The expected and the actual funding magnitudes were then compared. In univaritate regression analyses, we also evaluated the association of funding levels with changes of disease burden during 1990 and 2010, and with differences of disease burden between China and the worldwide average. The SPSS statistical package (Version 16, Chicago, Ill) was used for all statistic analyses.

## Results

The total NSFC budget of 2012 fiscal year was 16.93 billion RMB (US $2.69 billion), with 4.12 billion RMB allocated to health research (disease targeted), of which 2.57 billion RMB (62.4%) was devoted to the 53 diseases included in this study. Disease funding ranged from about 2 million RMB for multiple myeloma, to 328 million for leukemia, with a median of 31 million RMB (4.8±5.5 million RMB, **Table 1**).

**Table 1:** Funding levels of NSFC and disease burden in China.

Condition or disease		NSFC research fund	Measure of disease burden in 2010					
			thousands (rank)					
		thousands in RMB (% of total)	Mortality	Mortality change since 1990	YLLs	YLLs change since 1990	DALYs	DALYs change since 1990
Leukaemia		327854 (12.8)	58 (17)	5 (25)	2390 (15)	−366 (40)	2418 (19)	−354 (42)
Diabetes		176635 (6.9)	160 (11)	90 (5)	3173 (13)	1251 (5)	7835 (9)	2989 (4)
Ischemic heart disease		169185 (6.6)	949 (2)	499 (1)	16084 (3)	7093 (1)	17886 (3)	7759 (1)
Dementia		115785 (4.5)	50 (20)	24 (11)	616 (30)	159 (17)	1593 (23)	636 (13)
Liver cancer		114225 (4.5)	370 (6)	131 (4)	10014 (6)	2684 (4)	10089 (7)	2718 (5)
Injury		103560 (4.0)	796 (4)	−56 (47)	31759 (1)	−12827 (51)	40804 (1)	−10386 (51)
Breast cancer		95360 (3.7)	53 (19)	23 (12)	1563 (17)	645 (8)	1671 (22)	716 (10)
Asthma		80060 (3.1)	20 (32)	−32 (45)	358 (37)	−173 (38)	1095 (30)	−143 (39)
Colorectal cancer		75990 (3.0)	150 (12)	57 (6)	3305 (11)	901 (7)	3423 (15)	975 (8)
Chronic kidney diseases		75389 (2.9)	82 (15)	28 (9)	2054 (16)	413 (11)	2782 (17)	696 (11)
Cirrhosis		68085 (2.7)	114 (13)	−61 (48)	3180 (12)	−2095 (47)	3316 (16)	−2088 (47)
Neonatal disorders		63730 (2.5)	83 (14)	−260 (52)	7173 (7)	−22334 (53)	8679 (8)	−22534 (53)
Lung cancer		63580 (2.5)	513 (5)	253 (3)	11197 (5)	4943 (2)	11318 (6)	5013 (3)
Hypertensive heart disease		61463 (2.4)	173 (10)	39 (7)	2734 (14)	458 (10)	2767 (18)	460 (15)
Stroke		55330 (2.2)	1727 (1)	387 (2)	29173 (2)	4829 (3)	30139 (2)	5262 (2)
Stomach cancer		53610 (2.1)	297 (7)	0 (38)	6523 (8)	−849 (43)	6616 (10)	−825 (43)
Hepatitis		53245 (2.1)	44 (23)	11 (16)	1319 (19)	−48 (34)	1443 (28)	−38 (37)
Brain cancers		52610 (2.1)	49 (21)	13 (14)	1473 (18)	232 (14)	1497 (26)	240 (19)
Depression		51335 (2.0)	0 (53)	0 (36)	0 (53)	0 (30)	11767 (5)	2297 (6)
Pneumonia		46600 (1.8)	195 (8)	−197 (51)	4771 (9)	−20173 (52)	5135 (11)	−20166 (52)
Schizophrenia		43260 (1.7)	9 (42)	−4 (40)	279 (39)	−171 (37)	3472 (14)	918 (9)
HIV-AIDS		40075 (1.6)	36 (24)	36 (8)	1111 (23)	1100 (6)	1752 (20)	1739 (7)
Peptic ulcer		39870 (1.6)	19 (33)	−36 (46)	382 (35)	−942 (45)	453 (41)	−958 (45)
Atrial fibrillation and flutter		39150 (1.5)	13 (37)	8 (18)	154 (47)	91 (22)	596 (39)	259 (18)
Prostate cancer		34530 (1.3)	11 (39)	7 (19)	165 (45)	97 (21)	178 (50)	109 (25)
Nasopharynx cancer		31250 (1.2)	34 (26)	12 (15)	1046 (24)	314 (13)	1060 (31)	320 (17)
Epilepsy		31240 (1.2)	12 (38)	−7 (42)	559 (32)	−424 (41)	1507 (25)	−216 (40)
Maternal disorders		30260 (1.2)	5 (49)	−16 (43)	272 (40)	−911 (44)	546 (40)	−855 (44)
COPD		29450 (1.2)	934 (3)	−493 (53)	12995 (4)	−10808 (50)	16724 (4)	−9746 (50)
Parkinson disease		29160 (1.1)	7 (47)	3 (30)	121 (49)	25 (28)	265 (44)	91 (28)
Tuberculosis		26710 (1.0)	45 (22)	−124 (50)	1172 (22)	−4059 (49)	1733 (21)	−4418 (49)
Cervical cancer		26245 (1.0)	25 (29)	5 (26)	727 (28)	180 (16)	742 (35)	185 (21)
Pancreatic cancer		24051 (0.9)	58 (16)	26 (10)	1315 (20)	551 (9)	1322 (29)	555 (14)
Ovarian cancer		22270 (0.9)	21 (31)	6 (23)	594 (31)	141 (19)	603 (38)	145 (23)
Mouth cancer		19970 (0.8)	14 (36)	7 (21)	351 (38)	150 (18)	361 (43)	156 (22)
Oesophageal cancer		19480 (0.8)	176 (9)	7 (20)	3824 (10)	−15 (32)	3858 (12)	−10 (34)
Multiple sclerosis		18085 (0.7)	2 (51)	−2 (39)	50 (51)	−60 (35)	149 (52)	−26 (36)
Lymphoma		16400 (0.6)	29 (28)	6 (22)	835 (26)	40 (26)	854 (33)	50 (31)
Malaria		15050 (0.6)	0 (52)	0 (37)	2 (52)	−6 (31)	12 (53)	−4 (33)
Schistosomiasis		14650 (0.6)	9 (43)	0 (35)	222 (42)	−34 (33)	251 (45)	−23 (35)
Bladder cancer		13280 (0.5)	23 (30)	8 (17)	399 (34)	106 (20)	411 (42)	115 (24)
Pancreatitis		13110 (0.5)	8 (44)	2 (32)	188 (44)	25 (29)	205 (48)	27 (32)
Anaemias		12860 (0.5)	15 (35	−5 (41)	420 (33)	−494 (42)	1513 (24)	−346 (41)
Cardiomyopathy and myocarditis		12710 (0.5)	35 (25)	3 (31)	985 (25)	−125 (36)	1013 (32)	−127 (38)
Alcohole Chinae disorders		10720 (0.4)	5 (50)	1 (33)	163 (46)	32 (27)	3489 (13)	688 (12)
Kidney cancer		10020 (0.4)	32 (27)	18 (13)	823 (27)	400 (12)	837 (34)	384 (16)
Melanoma		8330 (0.3)	7 (48)	4 (28)	153 (48)	75 (24)	156 (51)	77 (29)
Gallbladder cancer		7560 (0.3)	18 (34)	0 (34)	637 (29)	225 (15)	644 (37)	229 (20)
Peripheral vascular disease		6990 (0.3)	9 (41)	6 (24)	119 (50)	−346 (39)	182 (49)	100 (26)
Thyroid cancer		6660 (0.3)	8 (45)	3 (29)	201 (43)	67 (25)	213 (47)	76 (30)
Meningitis		5350 (0.2)	8 (46)	−23 (44)	358 (36)	−1488 (46)	675 (36)	−1555 (46)
Rheumatic heart disease		4470 (0.2)	57 (18)	−74 (49)	1270 (21)	−2581 (48)	1487 (27)	−2654 (48)
Multiple myeloma		1710 (0.1)	9 (40)	4 (27)	246 (41)	90 (23)	250 (46)	93 (27)
**Association with NSFC research fund**	R		0.33	0.22	0.39	−0.04	0.40	−0.003
	P		0.02	0.12	0.004	0.79	0.003	0.98

In the univariate regression analysis, NSFC health funding was associated with burden of disease measured in mortality (R = 0.33, P = 0.02), YLLs (R = 0.39, P = 0.004), and DALYs (R = 0.40, P = 0.003, **Table 1**).

Since 1990, the burdens decreased in some diseases, while increased in others in large extents (**Table 1**). The mortality of chronic obstructive pulmonary disorder (COPD) decreased from 1427 thousand in 1990 to 934 thousand in 2010, with a decrement of 493 thousand which ranked top among diseases. The mortality of neonatal disorders decreased from 343 thousand to 83 thousand, with a decrement of 260 thousand which ranked second among diseases. Progress in neonatal disorders contributed the largest reduction of DALYs (a decrement of 22534), followed by pneumonia (a decrement of 20166). On the other hand, ischemic heart disease induced the largest increment of mortality (499 thousand) during 1990 to 2010, followed by stroke (387 thousand). Ischemic heart disease also induced the largest increment of DALYs (7759 thousand), followed by stroke (5262 thousand). Because these transitional trends of disease burden may be a reflection of past research strength, and could have been used as indicators for allocating research resources, we analyzed the changes of disease burdens during 1990 to 2010. But none of the changes of mortality (R = 0.22, P = 0.12), YLLs (R = −0.04, P = 0.79) and DALYs (R = −0.003, P = 0.98) was associated with NSFC funding magnitudes (**Table 1**).

Some diseases pose extra burden in China compare to the world averages, while others pose less burden. Of note, stroke demonstrated the largest extra mortality (126.1/100000 higher), YLLs (614/100000 higher) and DALYs (618/100000 higher) than the world averages. Ischemic heart disease induced the least extra mortality than the world average (35.6/100000 lower), and neonatal disorders induced the least extra YLLs (1985/100000 lower) and DALYs (2004/100000 lower) than the world averages. We analyzed differences of disease burden metrics in 2010 between China and world averages. But none of the differences of mortality (R = −0.11, P = 0.45), YLLs (R = −0.11, P = 0.43) and DALYs (R = −0.12, P = 0.38) to the world averages were associated with NFSC funding magnitudes (**Table 2**).

**Table 2:** Funding levels of NSFC and disease burden of world.

Condition or disease		NSFC research fund	Measure of disease burden in 2010					
			Age-standardized rate per 100000 (rank)					
		thousands in RMB (% of total)	Mortality,	Mortality,	YLLs,	YLLs,	DALYs,	DALYs,
			World	China-World	World	China-World	World	China-World
Leukaemia		327854 (12.8)	4.2(27)	0.0 (12)	137(23)	45 (7)	139(31)	46 (7)
Diabetes		176635 (6.9)	19.5(9)	−8.0 (46)	378(13)	−160 (43)	680(11)	−148 (41)
Ischemic heart disease		169185 (6.6)	105.7(1)	−35.6 (53)	1757(3)	−636 (48)	1884(3)	−642 (48)
Dementia		115785 (4.5)	7.1(18)	−3.2 (39)	66(34)	−20 (27)	165(28)	−46 (33)
Liver cancer		114225 (4.5)	11.5(14)	13.5 (4)	275(14)	379 (2)	277(17)	382 (2)
Injury		103560 (4.0)	74.3(3)	−17.6 (49)	3347(1)	−1024 (50)	4058(1)	−1118 (50)
Breast cancer		95360 (3.7)	6.6(19)	−3.1 (38)	161(21)	−62 (36)	174(27)	−67 (35)
Asthma		80060 (3.1)	5.2(23)	−3.7 (42)	125(27)	−100 (41)	326(15)	−242 (44)
Colorectal cancer		75990 (3.0)	10.8(17)	−0.2 (18)	201(19)	22 (9)	209(25)	21 (9)
Chronic kidney diseases		75389 (2.9)	11.1(16)	−5.3 (43)	249(15)	−108 (42)	307(16)	−116 (39)
Cirrhosis		68085 (2.7)	15.6(12)	−7.9 (45)	441(11)	−233 (45)	450(13)	−233 (43)
Neonatal disorders		63730 (2.5)	31.0(6)	−21.6 (51)	2794(2)	−1985 (53)	2931(2)	−2004 (53)
Lung cancer		63580 (2.5)	23.4(7)	12.4 (5)	465(10)	287 (3)	470(12)	290 (3)
Hypertensive heart disease		61463 (2.4)	13.1(13)	−0.3 (19)	216(17)	−23 (29)	222(23)	−28 (27)
Stroke		55330 (2.2)	88.4(2)	38.5 (1)	1421(5)	614 (1)	1484(5)	618 (1)
Stomach cancer		53610 (2.1)	11.5(15)	17.3 (3)	235(16)	204 (5)	238(20)	207 (4)
Hepatitis		53245 (2.1)	4.6(26)	−1.6 (33)	185(20)	−97 (40)	192(26)	−95 (37)
Brain cancers		52610 (2.1)	3(33)	0.3 (9)	87(32)	14 (10)	88(37)	14 (10)
Depression		51335 (2.0)	0(53)	0.0 (14)	0(53)	0 (16)	1078(9)	−260 (45)
Pneumonia		46600 (1.8)	41(5)	−25.1 (52)	1639(4)	−1204 (52)	1672(4)	−1208 (52)
Schizophrenia		43260 (1.7)	0.3(50)	0.3 (10)	9(49)	10 (12)	218(24)	8 (11)
HIV-AIDS		40075 (1.6)	21.4(8)	−19.0 (50)	1121(7)	−1010 (49)	1184(7)	−1065 (49)
Peptic ulcer		39870 (1.6)	3.7(29)	−2.3 (36)	93(28)	−67 (37)	98(33)	−67 (34)
Atrial fibrillation and flutter		39150 (1.5)	1.7(40)	−0.7 (25)	17(46)	−6 (21)	52(41)	−10 (21)
Prostate cancer		34530 (1.3)	3.8(28)	−2.9 (37)	48(38)	−36 (31)	55(39)	−42 (30)
Nasopharynx cancer		31250 (1.2)	1(46)	1.3 (7)	29(43)	39 (8)	29(47)	40 (8)
Epilepsy		31240 (1.2)	2.6(34)	−1.7 (35)	126(26)	−84 (39)	253(19)	−141 (40)
Maternal disorders		30260 (1.2)	3.7(30)	−3.4 (40)	208(18)	−189 (44)	234(21)	−196 (42)
COPD		29450 (1.2)	43.8(4)	26.8 (2)	687(8)	250 (4)	1114(8)	77 (6)
Parkinson disease		29160 (1.1)	1.7(41)	−1.2 (30)	19(45)	−11 (23)	28(48)	−9 (20)
Tuberculosis		26710 (1.0)	18(10)	−14.9 (47)	619(9)	−538 (47)	717(10)	−599 (47)
Cervical cancer		26245 (1.0)	3.4(32)	−1.7 (34)	92(30)	−45 (34)	94(35)	−46 (31)
Pancreatic cancer		24051 (0.9)	4.7(25)	−0.7 (24)	89(31)	−1 (18)	89(36)	−1 (15)
Ovarian cancer		22270 (0.9)	2.4(37)	−1.0 (28)	59(35)	−20 (26)	60(38)	−21 (25)
Mouth cancer		19970 (0.8)	1.9(39)	−0.9 (27)	45(39)	−22 (28)	47(43)	−23 (26)
Oesophageal cancer		19480 (0.8)	6.1(20)	6.2 (6)	129(24)	128 (6)	130(32)	129 (5)
Multiple sclerosis		18085 (0.7)	0.3(51)	−0.2 (17)	8(50)	−5 (20)	16(51)	−6 (18)
Lymphoma		16400 (0.6)	3.5(31)	−1.4 (31)	93(29)	−33 (30)	94(34)	−34 (29)
Malaria		15050 (0.6)	16.7(11)	−16.7 (48)	1141(6)	−1141 (51)	1200(6)	−1199 (51)
Schistosomiasis		14650 (0.6)	0.2(52)	0.4 (8)	5(52)	10 (11)	48(42)	−31 (28)
Bladder cancer		13280 (0.5)	2.6(35)	−0.9 (26)	42(41)	−14 (24)	44(45)	−15 (22)
Pancreatitis		13110 (0.5)	1.2(44)	−0.6 (23)	31(42)	−19 (25)	34(46)	−20 (24)
Anaemias		12860 (0.5)	1.7(42)	−0.5 (22)	79(33)	−46 (35)	227(22)	−105 (38)
Cardiomyopathy/myocarditis		12710 (0.5)	6.1(21)	−3.6 (41)	156(22)	−84 (38)	162(29)	−87 (36)
Alcohole Chinae disorders		10720 (0.4)	1.7(43)	−1.4 (32)	55(36)	−45 (33)	256(18)	−20 (23)
Kidney cancer		10020 (0.4)	2.5(36)	−0.3 (20)	52(37)	5 (13)	53(40)	4 (12)
Melanoma		8330 (0.3)	0.7(47)	−0.2 (16)	16(47)	−6 (22)	17(50)	−6 (19)
Gallbladder cancer		7560 (0.3)	2.3(38)	−0.2 (15)	44(40)	0 (17)	44(44)	0 (14)
Peripheral vascular disease		6990 (0.3)	0.7(48)	0.0 (13)	8(51)	0 (15)	14(52)	−1 (16)
Thyroid cancer		6660 (0.3)	0.5(49)	0.1 (11)	11(48)	2 (14)	12(53)	2 (13)
Meningitis		5350 (0.2)	6.1(22)	−5.5 (44)	389(12)	−357 (46)	426(14)	−372 (46)
Rheumatic heart disease		4470 (0.2)	5.2(24)	−1.2 (29)	127(25)	−41 (32)	147(30)	−46 (32)
Multiple myeloma		1710 (0.1)	1.1(45)	−0.4 (21)	21(44)	−4 (19)	21(49)	−4 (17)
**Association with NSFC research fund**	R		0.33	−0.11	0.24	−0.11	0.25	−0.12
	P		0.02	0.45	0.09	0.43	0.07	0.38

**Figure 1:**
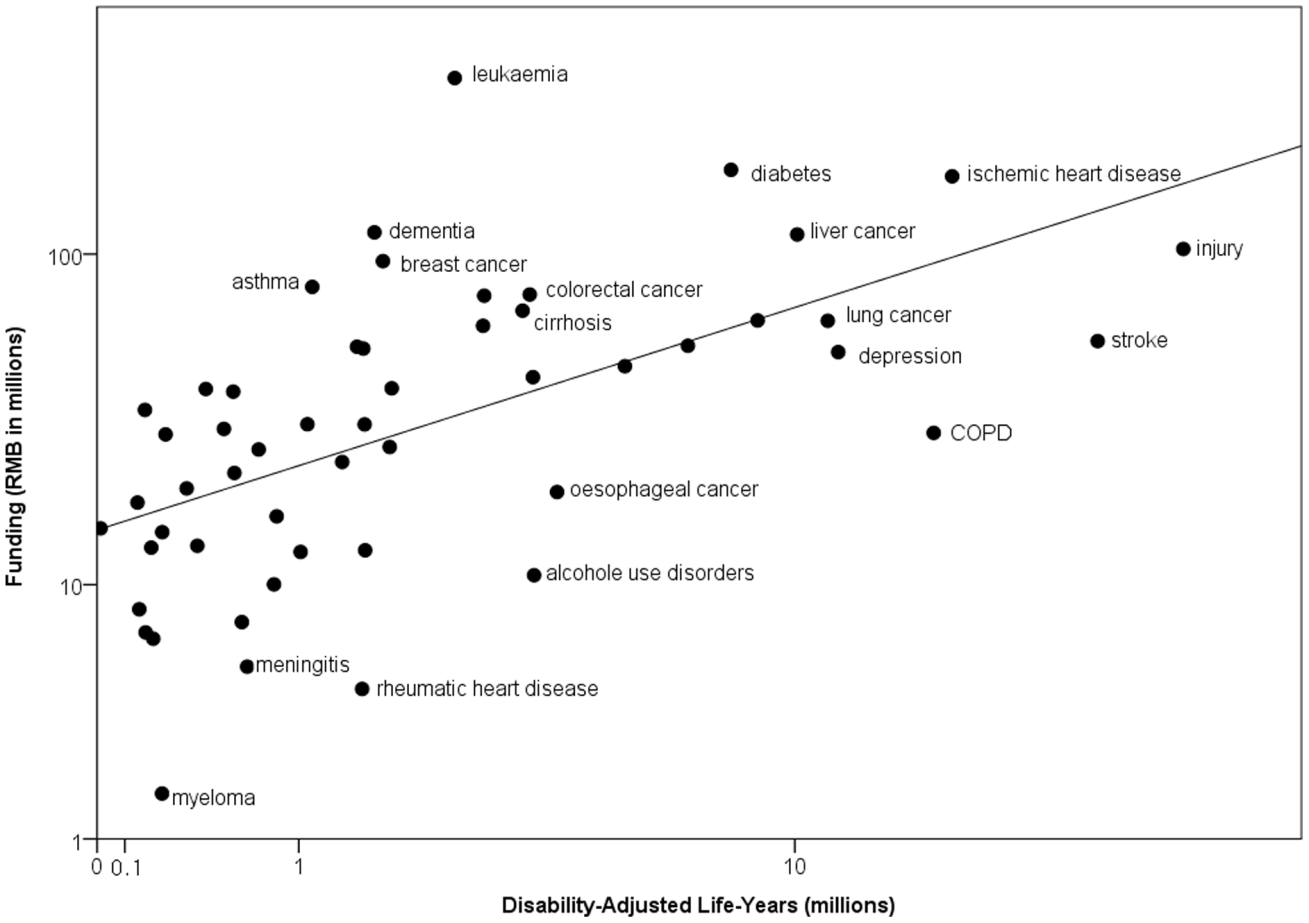
Relationship between NSFC disease-specific funding in 2012 and disability-adjusted life-years (DALYs) in 2012. Both X and Y axes are submitted to logarithmic scale. The line represents funding predicted on the basis of a linear regression with DALYs.

In standard multivariable analysis, DALYs became the only significant predictor for NSFC funding. The amount of predicted and actual NSFC funding as a function of DALYs is shown in **Figure 1**. The line represents the predicted funding levels if DALYs were the sole criterion for funding.

**Figure 2:**
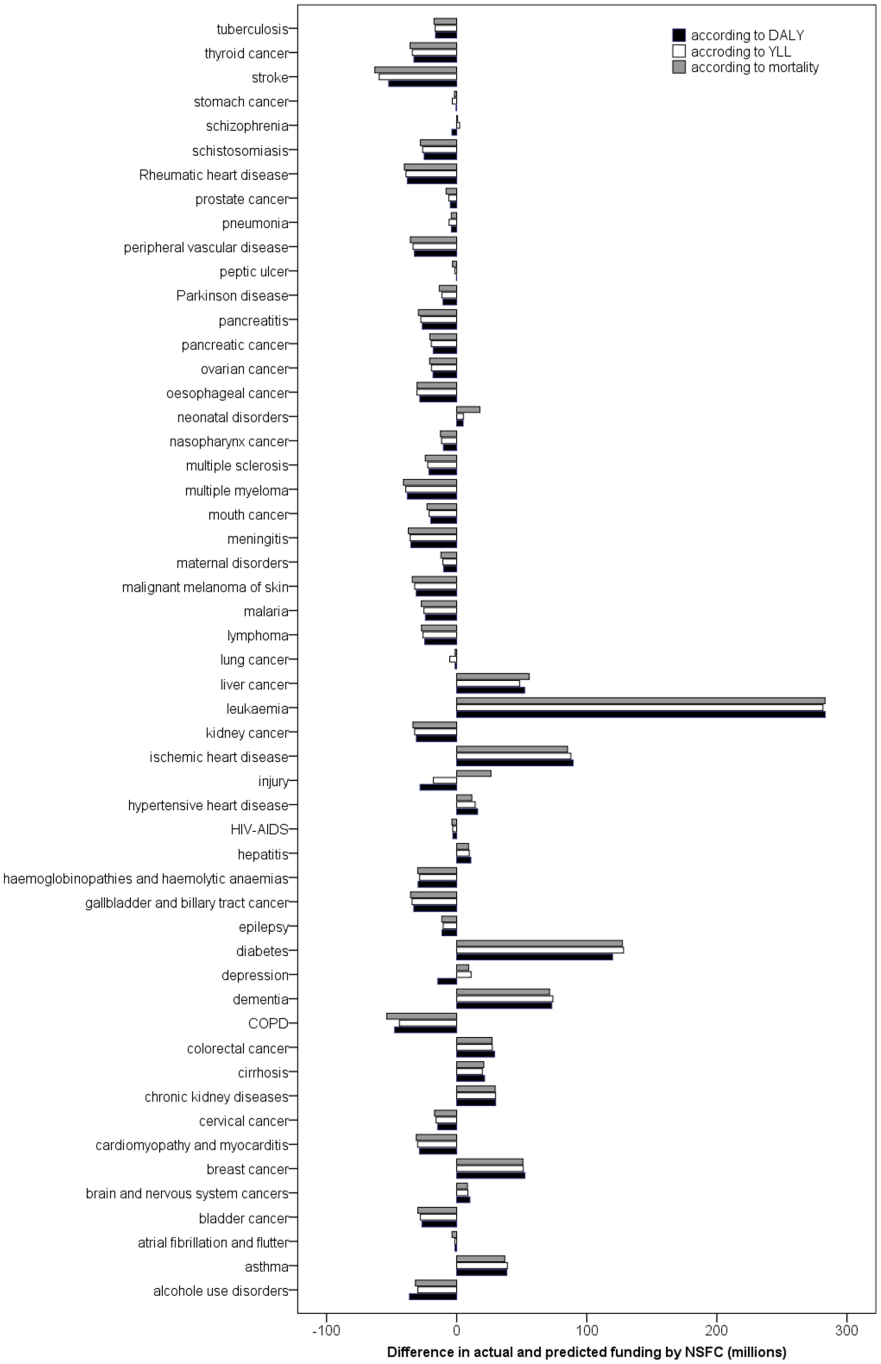
Differences between actual and expected disease-specific NSFC funding as predicted by mortality, YLLs and DALYs. Negative values indicates underfunded, and positive values indicates overfunded.

The difference between actual and predicted funding for each disease, with mortality, YLLs and DALYs as explanatory variables, is shown in **Figure 2**. Stroke, and COPD received the least funding compared to expected, while the leukemia and diabetes received the most funding compared to expected.

## Discussion

In this study we analyzed the relationship between NSFC funding and burden of disease measured in mortality, YLLs and DALYs. Although the NSFC health funding was associated with disease burden, measured in mortality (R = 0.33, P = 0.02), YLLs (R = 0.39, P = 0.004), and DALYs (R = 0.40, P = 0.003), there exist significant differences between the actual and expected funding in some diseases according to these measures.

The relationship between disease research funding and burden of disease has been investigated in US National Institute of Health (NIH) system in several studies [6], [7], [14]. Disease incidence and prevalence were unrelated to funding, while mortality and YLLs weakly correlated with funding. DALYs were more strongly predictive [6], [7]. In this study, mortality also weakly correlated with funding. Both YLLs and DALYs correlated significantly with NSFC funding. Although NSFC has a smaller total funding budget for health research, and is more inclusive and involves several subjects other than health, its organization is roughly similar to those of NIH. So it is not surprise that the overall profiles of NSFC funding distribution among diseases are similar to that of NIH.

The funding programs in NSFC can be classified as three types: free-applying, predetermined and investigator-targeted programs, which accounts for about 93%, 5% and 2% of the total budget respectively. For free-applying programs, there is no preset research scope, so applicants can choose any health research topics. For predetermined programs, every year one or two research topics were preset in each institute according to expertise and scientific needs. For the investigator-targeted programs, prominent investigators younger than 45 years were selected base on their previous academic achievements, and 1.5–3.0 million RMB was arranged to each awarder. With the money, they can choose any health research topics in five years. These arrangements made the distribution of NSFC health research fund among diseases largely determined by numbers of applications and applicants' interests in individual disease, which may be further influenced by international research trends, governmental policies and public opinions.

Based on the limited data of the present study, it is unlikely to determine why some diseases were underfunded, while others overfunded relative to disease burden. The over-funding of leukemia in China may be explained by the governmental reinforce of children health after the implementation of One-Child Policy in 1978. Successes in treating some types of leukemia in resent years, arsenic trioxide for acute promyelocytic leukemia as an example, which was originally discovered by Chinese researchers and they also contributed remarkably in the subsequent studies [15], [16], may have further incited the enthusiasm for leukemia research in China. On the other hand, stroke and COPD remained as the most underfunded diseases. The limited data suggested that incidence, prevalence of stroke, COPD and several other chronic diseases are increasing rapidly in China in recent year due to population ageing, lifestyle transition and environment pollution [17], [18]. These rapid changes in disease burdens have not reflected in the current NSFC funding assignment, possibly due to the unawareness of impact of these changes to public health or delayed responses to these changes. Furthermore, lack of major progress in managing stroke and COPD in recent years probably have depressed the interests for studying these chronic diseases.

Given the limited resource for health research, developing countries like China should allocate more research fund to their country-specific diseases–those with extra burden than other countries [19]. But in analyzing NCSF allocation, we failed to find this association. Measured by mortality, YLLs or DALYs, stroke poses the heaviest disease burden in China. The mortality, YLLs and DALYs of stroke in China is 38.5/100000, 614/100000 and 618/100000 higher than the world average level respectively, and all of these differences rank first among diseases. On the other hand, mortality, YLLs and DALYs of ischemic heart disease is 35.6/100000, 636/100000 and 642/100000 lower than the world average level respectively, which make it one of the least country-specific diseases in China. Although DALYs caused by stroke in 2010 (30139 thousand) is about two times of DALYs caused by ischemic heart disease (17886 thousand), the NSFC fund allocated for stroke is only one third of that for ischemic heart disease (55 vs 169 million RMB). This phenomenon of disproportionally underfunding of stroke and overfunding of ischemic heart disease in reference to disease burden was not observed in the recent NIH fund allocation [7].

The disease spectrum in China has changed significant in recent 20 years. Measured by DALYs, the disease burden of neonatal disorders and pneumonia decreased with the largest extent (22534/100000 and 20166/100000) during 1990 to 2010; while DALYs due to ischemic heart disease and stroke increased with the largest extent (7759/100000 and 5262/100000) [9]. Theoretically, those with disease burden increased rapidly deserve more research resources to find approach for counteracting the trends. But we failed to find this association in NCSF allocation.

There are several limitations in this study which should be addressed when interpreting the results. First, we did not evaluate other sources, such as health research funds from government agencies other than NSFC. Other national funds, such as 863 projects, 973 projects, and National Supporting Programs administrated by Ministry of Science and Technology (MOST), account for about 10% of all governmental funds dedicated to health research in 2012 [20]. Funds from provincial government account for about 20% of all governmental funds dedicated to health research. Thus, contribution of resources other than NSFC for health research is unremarkable. Second, we did not include prevalence and incidence as measures for disease burden due to lack of recent data. In the previous studies, prevalence and incidence were not associated with NIH funding levels. We presume this is also the case in NSFC funding. Third, there may be fluctuation of funding magnitudes for a given diseases over years. The annual fluctuation of funding may be more predominant in less common diseases, for which the number of applicants is relatively small.

In conclusion, although the funding levels of NSFC were roughly associated with diseases burden as measured with mortality, YLLs and DALYs, some major diseases such as stroke were underfunded; with others such as leukemia were overfunded. Changes of disease burden during the past 20 years, and differences of disease burden from the world average have not been considered in allocating the current NSFC health funds.
